# An Experimental Study on [^125^I]I-pHLIP (Var7) for SPECT/CT Imaging of an MDA-MB-231 Triple-Negative Breast Cancer Mouse Model by Targeting the Tumor Microenvironment

**DOI:** 10.1155/2021/5565932

**Published:** 2021-02-16

**Authors:** Mingming Yu, Yanqin Sun, Guangjie Yang, Zhenguang Wang

**Affiliations:** Department of Nuclear Medicine, The Affiliated Hospital of Qingdao University, Qingdao, China

## Abstract

**Objective:**

To evaluate the diagnostic efficacy of MDA-MB-231 triple-negative breast cancer with ^125^I-labeled pHLIP (Var7) by single-photon emission computed tomography/computed tomography (SPECT/CT) imaging.

**Methods:**

The binding fraction of [^125^I]I-pHLIP (Var7) and MDA-MB-231 cells was measured at pH 7.4 and pH 6.0, and tumor-bearing mice were subjected to small-animal SPECT/CT imaging studies.

**Results:**

At pH = 6.0, the binding fractions of [^125^I]I-pHLIP (Var7) and MDA-MB-231 cells at 10 min, 40 min, 1 h, and 2 h were 1.9 ± 0.1%, 3.5 ± 0.1%, 6.3 ± 0.8%, and 6.6 ± 0.3%, respectively. At pH = 7.4, there was no measured binding between [^125^I]I-pHLIP (Var7) and MDA-MB-231 cells. Small-animal SPECT/CT imaging showed clearly visible tumors at 1 and 2 h after injection.

**Conclusions:**

[^125^I]I-pHLIP (Var7) could bind to MDA-MB-231 cells in an acidic environment, and small-animal SPECT/CT imaging showed clear tumors at 1 and 2 h after probe injection.

## 1. Introduction

In solid malignancies, tumor cells grow in the tumor microenvironment (TME), the pH of which is acidic and can be as low as 6.0 [1–5]. Because the acidic TME is stable and not affected by the selection of tumor clones, an acidic TME is also considered a promising marker for tumor-targeted detection [4, 6]. Studies have shown that peptides of the pH (low) insertion peptide (pHLIP) family can target acidic tissues. When the extracellular environment is acidic, pHLIP inserts into the cell membrane spontaneously and forms a helical structure [7].

One study [8] successfully transported magnetic nanoparticles to MDA-MB-231 human breast cancer xenografts by using pHLIP, indicating that the MDA-MB-231 breast cancer model has an acidic microenvironment and that pHLIP can target acidic tissues. Labeling pHLIP with radionuclides can target tumor tissues, and radionuclides can be retained in tumors to allow sufficient time to remove nontumor-binding markers from normal tissues, thereby yielding high-contrast images [6, 9–13]. Currently, no study has investigated the labeling of pHLIP by radioactive iodine. This study attempted to label pHLIP with ^125^I to achieve single-photon emission computed tomography/computed tomography (SPECT/CT) imaging in an MDA-MB-231 triple-negative breast cancer mouse model. The labeling of pHLIP with ^125^I was used to investigate the following: (1) its usefulness for the SPECT imaging of tumor-bearing mice, tumor diagnosis, and therapeutic efficacy monitoring and as a preliminary preparation for positron emission tomography (PET) imaging performed with ^124^I-labeled probes. (2) The potential of using ^131^I-labeled probes for tumor therapy was evaluated by analyzing the distribution of radioactivity in tumor-bearing mice and the imaging results.

## 2. Materials and Methods

### 2.1. Main Experimental Instruments and Reagents

#### 2.1.1. Main Instruments

The main instruments used in this study included a gamma counter (GC-2016, Zhongke Zhongjia Scientific Instrument Co., Ltd., China), a radiopharmaceutical dose calibrator (CRC-55tR model, Capintec Inc., USA), and a small-animal SPECT/CT imaging system (U-SPECT +/CT model, MILabs, Netherlands).

#### 2.1.2. Main Reagents

The main reagents used in this study included [^125^I] NaI (McMaster University, Canada), chloramine-T (Sinopharm Chemical Reagent Co., Ltd., China), sodium metabisulfite (Sinopharm Chemical Reagent Co., Ltd.), and a C18 column (Waters, China).

### 2.2. Synthesis and Purification of pHLIP (Var7)

The pHLIP (Var7) sequence was synthesized by solid-phase peptide synthesis (SPPS) as follows: AEEQNPWARYLEWLFPTETLLLEL-NH_2_. Peptides were purified via a *Shimadzu LC*-20A series *high-performance liquid chromatography* (*HPLC*) instrument equipped with an Inertsil ODS-SP HPLC column (5 *μ*m, 4.6 × 250 mm) (*Shimadzu Corporation*, Japan) eluted with a gradient from 70 to 30% solvent A (0.1% trifluoroacetic in 100% water) and 0 to 100% solvent B (0.1% trifluoroacetic in 100% acetonitrile) at 1.0 ml/minute. The purity of the peptides was determined by HPLC, and their identities were confirmed via mass *spectrometric* analysis *using an LCMS-2020 series mass spectrometer* (*Shimadzu Corporation*, *Japan*).

### 2.3. ^125^I Labeling, Separation, and Purification

**Figure 1:**
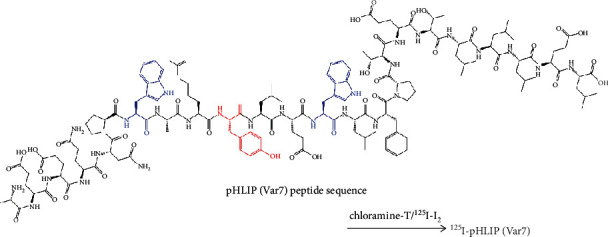
Schematic of ^125^I labeling of pHLIP (Var7). The red (tyrosine) and blue (tryptophan) dotted amino acids indicate the reactive amino acids where radioactive ^125^I may be attached.

pHLIP (Var7) was labeled with ^125^I by the chloramine-T method (Figure 1). One hundred micrograms of pHLIP (Var7) powder was dissolved in 20 *μ*L of DMSO, followed by the addition of phosphate-buffered saline (PBS) buffer (pH = 7.4) to obtain 200 *μ*L of pHLIP (Var7) solution. Then, 200 *μ*L of pHLIP (Var7) solution and 10 *μ*L of [^125^I] NaI (38.48 MBq) solution were thoroughly mixed, 20 *μ*L of 5 mg/mL chloramine-T solution was added, and the mixture was allowed to react at room temperature for 50 s. Next, 150 *μ*L of sodium metabisulfite solution (5 mg/mL) was added to extend the reaction for 5 min. The solution was passed through the activated C18 extraction column and rinsed first with distilled water and then with ethanol. The solution was blown with nitrogen to obtain a near-dry solution and then diluted with PBS buffer to obtain [^125^I]I-pHLIP (Var7). The radiochemical yield was then calculated.

The purity of [^125^I]I-pHLIP (Var7) was determined by paper chromatography. Normal saline was used as the developing agent, and Xinhua Grade 1 chromatography paper was used as the stationary phase. An appropriate amount of [^125^I]I-pHLIP (Var7) eluent was aspirated by a capillary pipette and spotted at the origin of the chromatographic filter paper. After drying, the paper was placed vertically into a test tube containing the developing agent; the unfolded chromatographic filter paper was removed, air dried, and cut at intervals of 1 cm. The radioactivity count was measured segment by segment to calculate the radiochemical purity.

### 2.4. Determination of In Vitro Stability

The purified [^125^I]I-pHLIP (Var7) was placed at 4°C, at room temperature (25°C) in PBS buffer, and at 37°C in mouse serum. The radiochemical purity at different time points was measured, and the stability was determined.

1. Two hundred microliters of purified [^125^I]I-pHLIP (Var7) solution was placed at 4°C and at room temperature in PBS buffer. Radiochemical purity was determined immediately after purification and at 1, 2, 4, 8, and 24 h after purification
2. Fifty microliters of purified [^125^I]I-pHLIP (Var7) solution was mixed with 100 *μ*L of mouse serum and placed in a 37°C water bath. Radiochemical purity was determined immediately after mixing and at 1, 2, 4, 8, and 24 h after mixing

### 2.5. Detection of the Cell Binding Fraction

#### 2.5.1. Cell Culture

The human breast cancer cell line MDA-MB-231 was purchased from Shanghai Institute of Biochemistry and Cell Biology, Chinese Academy of Sciences and cultured in L-15 medium containing 10% fetal bovine serum at 37°C without CO_2_.

#### 2.5.2. Cellular Uptake Test

1. *Cell Plating*. After the MDA-MB-231 cells were grown to greater than 90% confluence, the cell concentration was adjusted to approximately 1.0 × 10^5^ cells/mL, 1 mL of the cell suspension was added to each well of a 12-well plate, and cells were cultured overnight at 37°C in a cell incubator.
2. *Drug Administration*. At the logarithmic growth phase, the cells were treated with drugs on the day after attachment; 3.7 kBq of [^125^I]I-pHLIP (Var7) (20 *μ*L volume) was added to each well. The pH inside the wells was adjusted to 7.4 and 6.0, yielding two groups of cells according to the pH value.
3. After 10 min, 40 min, 1 h, and 2 h of culture without CO_2_, the supernatant was aspirated into a tube and washed with PBS of the corresponding pH (pH 7.4 and 6.0) twice. The wash solution was also added to the supernatant tube. The cells were completely digested with trypsin, after which the cell suspension was aspirated into a tube and washed twice with PBS; the wash solution was also added to the tube. The radioactive counts of the cell tube (*B*) and supernatant tube (*F*) were measured by a *γ* counter. The cell binding fraction of the marker was calculated as *B*/(*B* + *F*) × 100%

### 2.6. Animal Experiments

#### 2.6.1. Establishment of the Animal Model

A mouse model bearing a human breast cancer cell line, MDA-MB-231, was established. MDA-MB-231 cells were cultured using the conventional method. Cells were collected at the logarithmic growth phase and washed once with PBS. The cells were then diluted to prepare suspensions at a concentration of approximately 1 × 10^6^ cells/mL, which were temporarily stored in an ice box. Female 4-week-old BALB/c nude mice (16-20 g) were selected. MDA-MB-231 cells were inoculated subcutaneously using a disposable insulin syringe inserted into the right lateral thighs of the nude mice. The number of inoculated cells in each nude mouse was approximately 0.1 mL. The nude mice were raised under specific pathogen-free (SPF) conditions.

#### 2.6.2. Biodistribution

Biodistribution experiments were conducted when the tumor diameter was 0.8-1.0 cm. Purified [^125^I]I-pHLIP (Var7) was diluted with PBS, and a total of 15 tumor-bearing nude mice with no significant differences in tumor size were randomly divided into five cohorts. Nude mice in each cohort were injected with [^125^I]I-pHLIP (Var7) at approximately 1480 kBq/200 *μ*L through the tail vein. At 0.5, 1, 2, 4, and 8 h after injection, blood was collected from the orbital sinus. The mice were sacrificed, and tissues and organs, including the heart, liver, spleen, lung, kidney, pancreas, stomach, small intestine, bladder, thyroid, brain, bone, muscle, and tumors, were collected and weighed. The radioactive count was measured, and the results were converted to %ID/g.

#### 2.6.3. Small-Animal SPECT/CT Imaging

Imaging was started when the diameter of the tumor grew to 0.8-1.0 cm. Three days before imaging, 3 nude mice began to drink 0.1% KI solution to block the thyroid gland until the end of the experiment.

1. The machine was calibrated daily
2. *Anesthesia Induction*. The tumor-bearing mice were anesthetized with isoflurane.
3. When the righting reflex of the tumor-bearing mice disappeared, 3.7 MBq/200 *μ*L of [^125^I]I-pHLIP (Var7) was injected into the tail vein
4. After [^125^I]I-pHLIP (Var7) injection, scanning was performed at 1, 2, 4, 8, and 24 h using static 10 min SPECT and medium-resolution whole-body CT. Imaging settings for the SPECT/CT and reconstruction are as follows: the time per frame was 10 min, voxel size was 0.8 mm, number of subsets was 32, and number of iterations was 15
5. The injection time, injection dose, and residual dose were recorded according to the tables in the experiment

### 2.7. Statistical Analysis

The SPSS 24.0.0.0 (IBM) statistical software was used for data processing. Variables are expressed as the mean (M) ± standard deviation (SD). One-way analysis of variance (ANOVA) was used for comparisons of variables. *P* values less than 0.05 were considered statistically significant.

## 3. Results

### 3.1. Design, Synthesis, and Purification of Peptides

**Figure 2:**
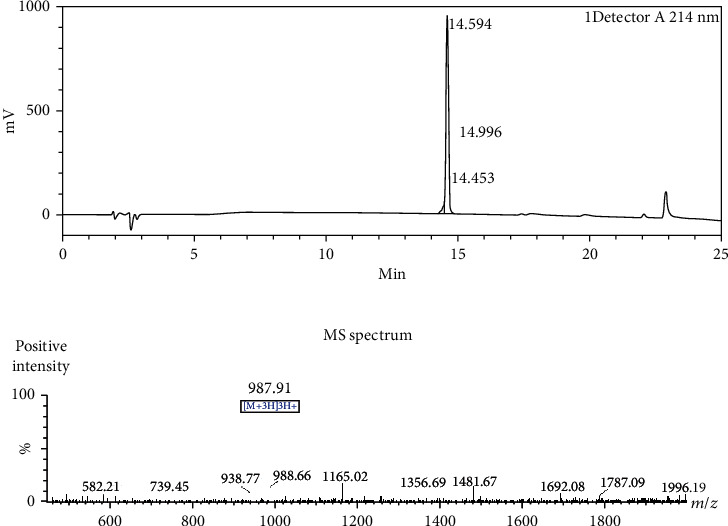
(a) HPLC and (b) MS analyses of pHLIP (Var7).

HPLC analysis of the product indicated the formation of several compounds, including a major product (95.1%, retention time: 14.6 min) and at least two smaller products at retention times of 14.5 and 15.0 min (Figure 2(a)). The mass spectrometry (ESI-MS) analysis of pHLIP (Var7) showed a mass peak with an *m*/*z* of 987.9 ([M + 3H]3H+) (Figure 2(b)). The experimentally observed molecular weight (2960.7) correlated well with the theoretical molecular weight (2961.3).

### 3.2. Radiochemical Yield and Radiochemical Purity

The radiochemical yield was 30.3 ± 2.2%, and the radiochemical purity was 98.6 ± 1.3%. The molar activity of [^125^I]I-pHLIP (Var7) was 345 ± 25 MBq/*μ*mol.

### 3.3. Determination of In Vitro Stability

**Figure 3:**
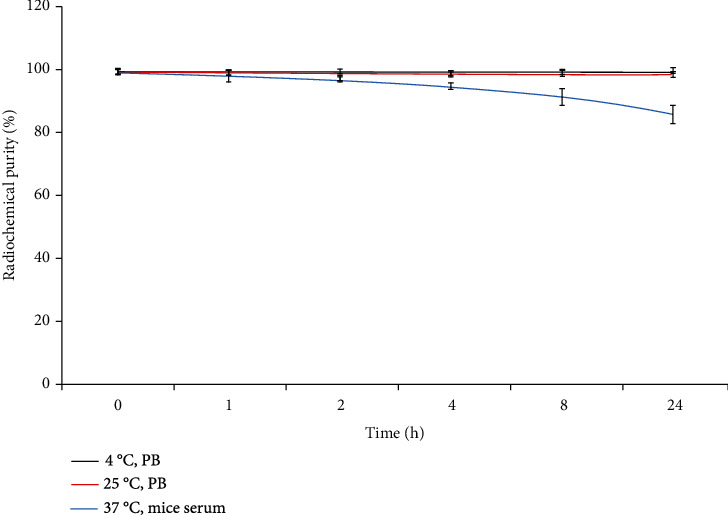
The stability of [^125^I]I-pHLIP (Var7) at 4°C, room temperature in PBS buffer, and 37°C in serum.

[^125^I]I-pHLIP (Var7) had a relatively high stability at 4°C and at room temperature in PBS buffer and was still higher than 98% at 24 h. The probe stabilities at 37°C in serum for 1, 2, 4, 8, and 24 h were 97.8 ± 1.9%, 96.8 ± 1.0%, 94.5 ± 1.1%, 91.3 ± 2.6%, and 85.6 ± 2.9%, respectively (Figure 3).

### 3.4. Determination of the Binding Fractions of ^125^I-pHLIP (Var7) to Cells at Different pH Values

**Figure 4:**
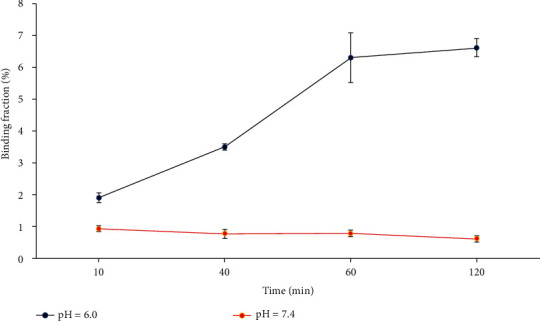
Determination of the affinity of [^125^I]I-pHLIP (Var7) for MDA-MB-231 cells.

At pH = 6.0, the binding fractions of [^125^I]I-pHLIP (Var7) and MDA-MB-231 cells at 10 min, 40 min, 1 h, and 2 h were 1.9 ± 0.1%, 3.5 ± 0.1%, 6.3 ± 0.8%, and 6.6 ± 0.3%, respectively. At pH = 7.4, the binding fractions of [^125^I]I-pHLIP (Var7) and MDA-MB-231 cells at 10 min, 40 min, 1 h, and 2 h were 0.9 ± 0.1%, 0.8 ± 0.1%, 0.8 ± 0.1%, and 0.6 ± 0.1%, respectively. At pH = 6.0, the cell binding fraction at each time point was significantly higher than that at pH = 7.4 (*P* < 0.001) (Figure 4).

### 3.5. Biodistribution

**Figure 5:**
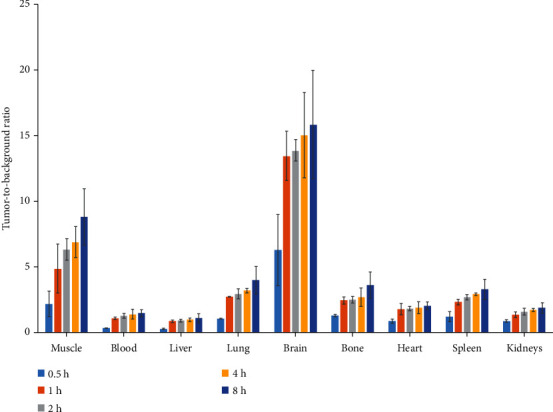
Tumor-to-background ratios of [^125^I]I-pHLIP (Var7) in mice with MDA-MB-231 tumors.

The distribution of [^125^I]I-pHLIP (Var7) in the tumors was highest (6.9 ± 0.4%ID/g) at 1 h after injection (*P* < 0.01) and then decreased with time, which were 3.6 ± 0.7, 2.0 ± 0.6, and 1.0 ± 0.4%ID/g at 2, 4, and 8 h, respectively. The distribution in the blood was 12.0 ± 1.5%ID/g at 0.5 h and decreased rapidly with time, which were 6.5 ± 1.5, 2.8 ± 0.6, 1.2 ± 0.5, and 0.5 ± 0.1%ID/g at 1, 2, 4, and 8 h, respectively. In addition, the distribution in the liver was relatively high, which were 13.9 ± 2.7, 8.0 ± 1.9, 4.0 ± 0.6, 1.8 ± 0.2, and 0.7 ± 0.2%ID/g at 0.5, 1, 2, 4, and 8 h, respectively (Table 1). The radioactive count ratios of tumors and major organs or tissues generally increased over time (Figure 5).

### 3.6. Small-Animal SPECT/CT Imaging

**Figure 6:**
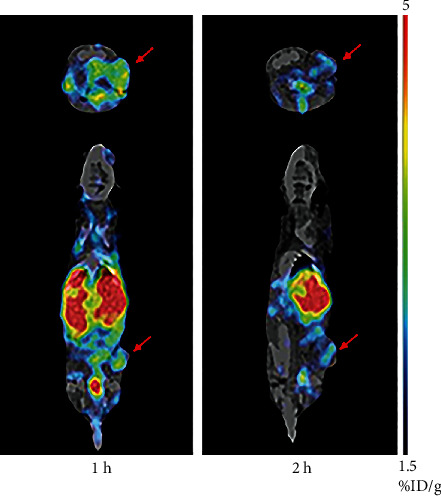
Small-animal SPECT/CT imaging of mice with MDA-MB-231 tumors (arrows).

The tumors were clearly visible at 1 and 2 h after the injection of [^125^I]I-pHLIP (Var7); the imaging result at 1 h was better than that at 2 h, and liver imaging was pronounced (Figure 6).

## 4. Discussion

Almost all solid tumors have an acidic TME with a pH as low as 6.0 [3–5]. The mechanisms of the acidic TME include hypoxia-induced aerobic glycolysis, aerobic glycolysis (Warburg effect), increased CO_2_ production due to uncontrolled cell growth, and increased ion pump activity on the cell membrane [1, 3]. Because the acidic TME is stable and not affected by the selection of tumor clones, an acidic TME is considered a promising marker for tumor-targeted detection [4, 6].

Studies have shown that pHLIP family peptides can sense the pH near the cell membrane and spontaneously insert into the cell membrane to form a helical structure when the extracellular environment is acidic [7]. The mechanism of action of pHLIP has been clarified. The entire hydrophobic intermediate region and C-terminal membrane insertion region of the peptide are negatively charged at physiological pH. As the pH value decreases, the peptide becomes neutral via protonation, and the loss of charge and the increase in total hydrophobicity cause pHLIP to form a transmembrane helix across the lipid bilayer. The N-terminus remains outside the cell, and the C-terminus remains within the cell to stably anchor the peptide on the cell membrane.

The pHLIP peptide family has broad prospects in tumor diagnosis and targeted therapy as follows. (1) pHLIP can connect the imaging agent to the cell surface and be used for tumor diagnostic imaging. Some studies [9, 10, 14–16] performed fluorescently labeled pHLIP imaging of tumor-bearing mouse models (breast cancer, prostate cancer, melanoma, and pancreatic cancer) and confirmed that these fluorescent probes had enhanced tumor-targeting abilities. Labeling pHLIP with radionuclides can favorably target tumor tissues with high image contrast [6, 9–13]. (2) In addition, pHLIP can transport different types of molecules, including small-molecule toxins, chemotherapeutic agents, protein fragments, peptide nucleic acids, pDNA, and nanoparticles, into cells for therapeutic purposes [17–33].

Four sequences in the pHLIP peptide family have enhanced tumor-targeting properties: wild type (WT), variant 3 (Var3), variant 7 (Var7), and ATRAM [34]. Var7 is the shortest and most polar peptide sequence and has the advantages of easy synthesis and fast blood clearance and was therefore selected Var7 as the study object. pHLIP (Var7) can be ^125^I-labeled by the chloramine-T method, and the labeling efficiency and radiochemical purity meet experimental requirements. Iodine binds to the tyrosine residue, which resides in the middle of the pHLIP (Var7) transmembrane domain and has the potential to affect insertion and adoption of the alpha-helical structure. However, ex vivo and in vivo results showed that this was not an issue. [^125^I]I-pHLIP (Var7) had a relatively high stability at 4°C and 25°C in PBS buffer and was still higher than 98% at 24 h, and the stability at 37°C in serum for 24 h was greater than 85%, which basically satisfied the imaging requirement. The relatively low stability at 37°C in serum may be related to deiodination activity or decomposition of the markers.

[^125^I]I-pHLIP (Var7) at pH = 6.0 had a significantly higher binding fraction with MDA-MB-231 cells than that at pH = 7.4. Under the condition of pH = 6.0, the binding fraction of [^125^I]I-pHLIP (Var7) and MDA-MB-231 cells increased with time, rapidly increasing to approximately 6.3% at 1 h and stabilizing at approximately 6.6% at 2 h. At pH = 7.4, [^125^I]I-pHLIP (Var7) and MDA-MB-231 essentially did not bind. This study suggests that [^125^I]I-pHLIP (Var7) can target MDA-MB-231 cells in an acidic environment.

[^125^I]I-pHLIP (Var7) showed the highest uptake value (6.9 ± 0.4%ID/g) in tumors at 1 h after injection into the body and then began to decrease. In addition, [^125^I]I-pHLIP (Var7) had a relatively high uptake in the thyroid, stomach, liver, and blood. [^125^I]I-pHLIP (Var7) showed some retention in the thyroid gland, suggesting that deiodination may occur in the body. Therefore, the thyroid gland must be blocked before imaging MDA-MB-231 tumor-bearing mice. Josefsson et al. [35] showed that free iodine was enriched in the stomach due to a high expression level of Na^+^/I^−^ symporters in the gastric mucosa. Therefore, the radioactive count of [^125^I]I-pHLIP (Var7) in the stomach of experimental animals may reflect iodine or iodine-labeled amino acid fragments detached from the markers. The radioactive distribution of the probe in the liver was significantly higher than that in the kidney, suggesting that the probe was mainly metabolized by the liver. The probe was mainly composed of nonpolar amino acids (13 nonpolar amino acids and 11 polar amino acids) with relatively poor water solubility, which is not conducive to excretion by the urinary system. The time-radioactivity relationship of [^125^I]I-pHLIP (Var7) in the mouse models revealed rapid blood clearance, with 12.0 ± 1.5%ID/g at 0.5 h after injection of 1480 kBq [^125^I]I-pHLIP (Var7), 1.2 ± 0.5%ID/g at 4 h, and 0.5 ± 0.1%ID/g at 8 h postinjection. The results of SPECT/CT imaging in small animals basically showed the same biodistribution trend, as liver uptake was the highest among all organs analyzed, and tumor uptake at 1 h was relatively high.

Some limitations exist in this study are as follows: (1) the probe had a relatively high blood background, which reduced image quality. Therefore, further optimization of the probe structure is necessary to increase its polarity so that probe not been bound to the tumor is excreted as soon as possible. (2) Another limitation was the low tumor/liver ratio, thus making it very difficult to detect breast cancer liver metastases by SPECT imaging. In addition, the use of ^131^I for probe labeling during internal radiation treatment of MDA-MB-231 breast cancer results in radiation injury to the liver. Although radioiodine-labeled pHLIP (Var7) has some disadvantages, the method is simple, and SPECT imaging can be used, at least to some extent, to diagnose and stage the tumor and evaluate the treatment efficacy; this method thus has a certain practical value.

## 5. Conclusion

This study found that ^125^I was successfully labeled with pHLIP (Var7) by the chloramine-T method, and [^125^I]I-pHLIP (Var7) could bind to MDA-MB-231 cells in an acidic environment. The biodistribution results showed that the probe had the highest radioactive distribution in the tumor at 1 h after injection. Tumor images were clearly observed in small animals by SPECT at 1 and 2 h after probe injection.

## Figures and Tables

**Table 1:** Distribution of [^125^I]I-pHLIP (Var7) in MDA-MB-231 tumor-bearing nude mice (%ID/g, *n* = 3).

Organs	Time (h)				
	0.5	1	2	4	8
Heart	4.1 ± 0.5	3.9 ± 0.7	2.0 ± 0.4	1.1 ± 1.1	0.5 ± 0.3
Liver	13.9 ± 2.7	8.0 ± 1.9	4.0 ± 0.6	1.8 ± 0.2	0.7 ± 0.2
Spleen	3.0 ± 1.1	3.0 ± 0.6	1.4 ± 0.4	0.7 ± 0.2	0.3 ± 0.1
Lung	3.4 ± 1.2	2.5 ± 1.0	1.2 ± 0.5	0.5 ± 0.3	0.2 ± 0.1
Kidney	4.2 ± 0.6	5.1 ± 1.1	2.3 ± 0.8	1.2 ± 0.4	0.5 ± 0.2
Pancreas	3.3 ± 0.8	4.1 ± 1.2	2.1 ± 0.6	0.9 ± 0.3	0.3 ± 0.1
Stomach	4.7 ± 1.2	5.1 ± 1.2	4.3 ± 1.0	2.2 ± 0.8	0.7 ± 0.2
Small intestine	3.3 ± 1.5	4.2 ± 0.6	2.8 ± 1.0	1.3 ± 0.5	0.4 ± 0.1
Bladder	3.8 ± 1.1	4.3 ± 1.1	2.1 ± 0.9	1.0 ± 0.6	0.5 ± 0.2
Thyroid	4.3 ± 1.7	5.6 ± 1.4	3.4 ± 1.0	1.6 ± 0.8	0.8 ± 0.4
Brain	0.6 ± 0.1	0.5 ± 0.1	0.3 ± 0.1	0.1 ± 0.1	0.1 ± 0.1
Bone	2.9 ± 0.3	2.8 ± 0.1	1.5 ± 0.5	0.7 ± 0.2	0.2 ± 0.1
Muscle	1.7 ± 0.6	1.4 ± 0.8	0.6 ± 0.2	0.2 ± 0.1	0.1 ± 0.1
Blood	12.0 ± 1.5	6.5 ± 1.5	2.8 ± 0.6	1.2 ± 0.5	0.5 ± 0.1
Tumor	3.6 ± 0.5	6.9 ± 0.4	3.6 ± 0.7	2.0 ± 0.6	1.0 ± 0.4

## Data Availability

The datasets used or analyzed during the current study are available from the corresponding author upon reasonable request.
